# Dr. Charles A. Osborne

**Published:** 1903-02

**Authors:** 


### OBITUARY.

Dr. Charles A. Osborne, U. of B. '61, of Owosso, Mich., died at his home January 2, 1903, aged 70 years. Dr. Osborne served as surgeon of the Eleventh Michigan Cavalry regiment in the civil war and became a physician of prominence at Owosso. He died of paralysis after a long illness.

